# Supplementary figures and images for: 3D printing of bioreactors in tissue engineering: A generalised approach
Source: PLoS One. 2020 Nov 30;15(11):e0242615. doi: 10.1371/journal.pone.0242615 (PMC7703892; doi:10.1371/journal.pone.0242615)

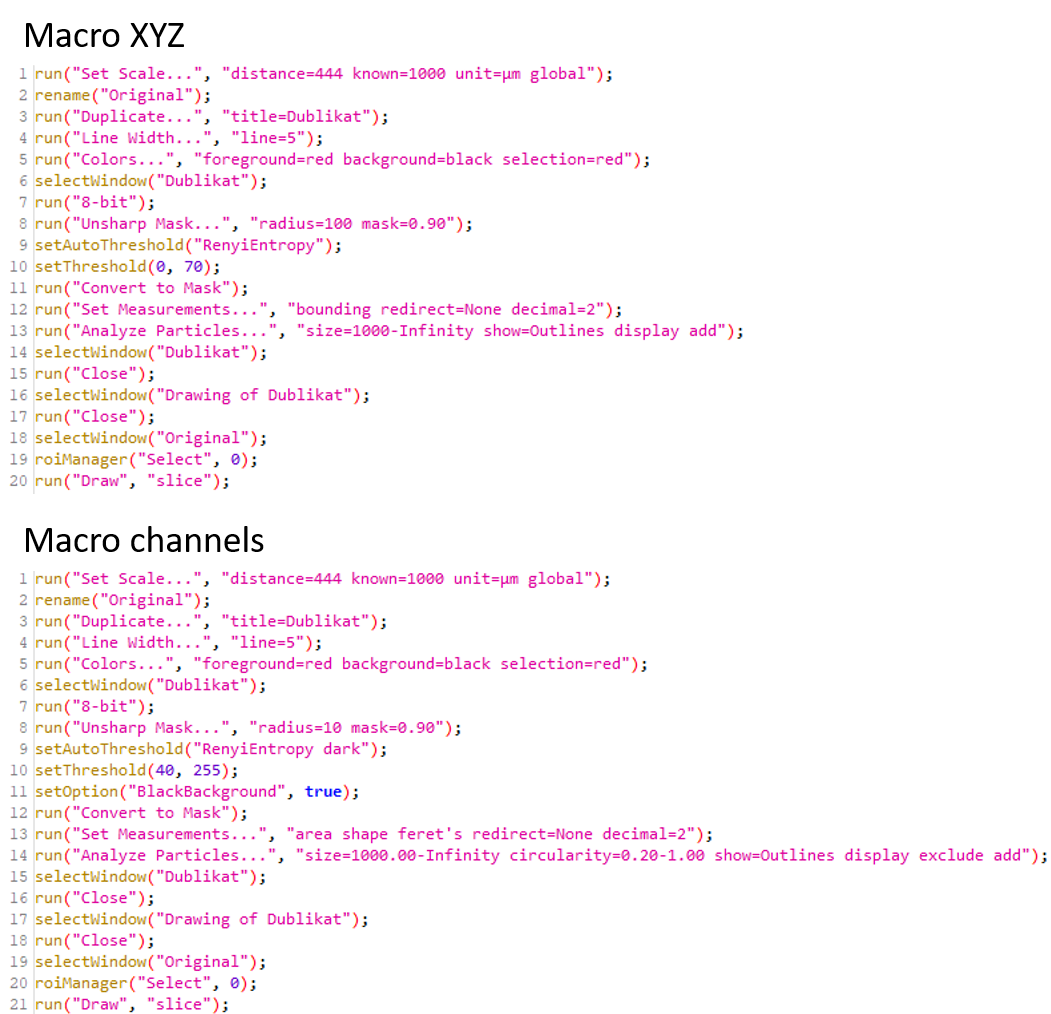

Supplement: S1 Fig — The resulting geometries of the printed test bodies were visualised by microscopic pictures and then analysed by the Fiji macros shown here. For quantification of the shape of the basic axes test bodies X, Y and Z the same macro was used (Macro XYZ). For quantification of the channels diameter and roundness a second macro was used (Macro channels). The area of interest was cropped before applying the macros. In both macros, the picture was set to 8-bit grayscale first. Following, an unsharp mask filter was applied to increase contrast. This filter was set to radius 100 (Macro XYZ), respectively 10 (Macro channels) and mask 0.9 for both. Then a grey level threshold (RenyiEntropy), set to 0/70 for Macro XYZ and 40/225 for Macro channels, was applied to reduce noise. Finally, the particle analyser tool identified the shape of the area of interest. Particle analyser was set to size 1000/infinity in both macros. Additionally, the circularity limit was set to 0.2/1 to ignore remaining artifacts in Macro channels. (TIF) [file pone.0242615.s001.tif]
