# Supplementary material for: 3D printing of bioreactors in tissue engineering: A generalised approach
Source: PLoS One. 2020 Nov 30;15(11):e0242615. doi: 10.1371/journal.pone.0242615 (PMC7703892; doi:10.1371/journal.pone.0242615)
Supplement: Attachment — Submitted filename: comment.doc [file pone.0242615.s005.doc]

In this manuscript, the authors tried to report a general approach to prepare the bioreactors via 3D printing for tissue engineering applications, not typical subtractive manufacturing methods. These work offer some summary and guideline information for details such as accuracy, cost, price-performance-effort ratio, post processing, product geometries, specific capabilities, limits of various additive manufacturing methods, which is also proofed and practiced by the corresponding experimental results via this guidance system.

Overall, this is a very extensive and well-organized study, which is also helpful to deeply identify a more suitable method for the manufacturing of a complex bioreactor system, so I recommend this work to be published in this journal.
