# Supplementary material for: 3D printing of bioreactors in tissue engineering: A generalised approach
Source: PLoS One. 2020 Nov 30;15(11):e0242615. doi: 10.1371/journal.pone.0242615 (PMC7703892; doi:10.1371/journal.pone.0242615)
Supplement: Attachment — Submitted filename: Response to Reviewers.docx [file pone.0242615.s006.docx]

To the Editorial Office – PLOS ONE

|  | **Bitte bei Antwort angeben:** | |  |
| --- | --- | --- | --- |
| Ihre Nachricht vom: | Unser Zeichen: | Telefon: +49 931 31-80183 | Würzburg, 02.11.2020 |
| Ihr Zeichen: |  | Telefax: +49 931 31 81068 |  |
| Fallnummer: |  | E-Mail: anna.stefl@uni-wuerzburg.de | |

Komm. Leitung
Prof. Dr. Jürgen Groll

Tel.: +49 931 201-73610

Sekretariat

Tel.: +49 931 31-80183

Fax: +49 931 31-81068

**3D printing of bioreactors in tissue engineering: A generalised approach**

Dear Dr. Yi Cao,

We hereby would like to submit the revision of our manuscript entitled: „3D printing of bioreactors in tissue engineering: A generalised approach”. We thank you very much for your assistance and handling of our manuscript.

Our manuscript has been carefully revised and we respond to the comments and suggestions of the reviewers point to point (see below).

We thank you and the reviewers for the valuable comments and the effort to improve the manuscript. Furthermore, we are grateful for the encouraging words from the reviewers.

For revision, we highlighted all changes. Additionally, we prepared a plain version of the manuscript.

All authors have approved this revised version.

Yours sincerely,

Marius Gensler

**Point-to-point responses**

**Editor:**

1) Please ensure that your manuscript meets PLOS ONE's style requirements, including those for file naming. The PLOS ONE style templates can be found at

https://journals.plos.org/plosone/s/file?id=wjVg/PLOSOne_formatting_sample_main_body.pdf and

https://journals.plos.org/plosone/s/file?id=ba62/PLOSOne_formatting_sample_title_authors_affiliations.pdf

Response:

Thank you for indicating problems within style and formatting. We have double-checked our manuscript to suit your formatting requirements.

2) We note that you have included the phrase “data not shown” in your manuscript. Unfortunately, this does not meet our data sharing requirements. PLOS does not permit references to inaccessible data. We require that authors provide all relevant data within the paper, Supporting Information files, or in an acceptable, public repository. Please add a citation to support this phrase or upload the data that corresponds with these findings to a stable repository (such as Figshare or Dryad) and provide and URLs, DOIs, or accession numbers that may be used to access these data. Or, if the data are not a core part of the research being presented in your study, we ask that you remove the phrase that refers to these data.

Response:

Line 376: The phrase “data not shown” was deleted. Within this paragraph, we explain our over-all guidance system. The data to which we referred, is valid for one specific process step (sterilisation) and material. Thus, it does not need to be included in the manuscript as the guidance system describes a general approach instead of a specific realisation.

Line 579: The phrase “data not shown” was deleted. In this paragraph, we list additional options to improve the printing process. These recommendations need to be tested individually by every user. Thus, it is not part of the actual research.

**Reviewer #1:**

In this manuscript, the authors tried to report a general approach to prepare the bioreactors via 3D printing for tissue engineering applications, not typical subtractive manufacturing methods. These work offer some summary and guideline information for details such as accuracy, cost, price-performance-effort ratio, post processing, product geometries, specific capabilities, limits of various additive manufacturing methods, which is also proofed and practiced by the corresponding experimental results via this guidance system.

Overall, this is a very extensive and well-organized study, which is also helpful to deeply identify a more suitable method for the manufacturing of a complex bioreactor system, I think it shoud be acceptable and I'd like recommend this work to be published in this journal.

Response:

Thank you for improving our manuscript by your revision. We are greatful to get this positive and encouraging feedback.

**Reviewer #2:**

The manuscript entitled “3D printing of bioreactors in tissue engineering: A generalised approach” was designed a guidance including test bodies to elucidate the real printing performance for a given printer system. In this manuscript, the authors have done a lot of very meaningful work, performance parameters such as the accuracy and mechanical stability of the test body are analyzed, and post-processing steps such as sterilization or cleaning are also considered.

Response:

Thank you very much for your encouraging feedback and the valuable advices. We appreciate the opportunity to improve our manuscript.

1) What problems should be paid attention to in the design of bioreactor?

Response:

We fully agree to the reviewers comment. There are many requirements and challenges when designing a bioreactor for tissue engineering applications which are extensively discussed in the literature and cited in the manuscript. We included the following sentence in the introduction (line 68-69):

“Further requirements and general concepts of bioreactor design have been discussed in the literature [23,25].”

Nevertheless, in the introduction (line 59-69) we describe the general requirements for bioreactor systems in tissue engineering. In the results (line 459-469), we point out for what purpose the printed bioreactor was designed and how this is reflected in the concept of the bioreactor.

To further emphasize a major problem in 3D printing of the bioreactors, we inserted two sentences regarding the discrepancy between the conceptualized and the printed part (606-608):

“A crucial problem is the discrepancy between the conceptualized and the printed parts. According to the gained knowledge about tolerances and limitations of the printer, the designed geometry of the bioreactor was adapted.”

We hope that we could clarify this comment sufficiently.

2) Please explain the difference and connection between bioreactor and bionic.

Response:

Line 78-79: We state, that 3D printing is much better suited to manufacture irregular shapes as they can be found in nature, compared to classic subtracting methods. These shapes offer the option to print more complex bioreactor geometries. We noticed, that the phrase “bionic” could be missleading, so we exchanged the phrase „bionic“ by „organic“ and adapted the sentence accordingly:

„These techniques are well suited for rapid prototyping of complex organic shapes, as they can be found in nature, and hollow geometries.”

3) As we all know, 3D printing still has many problems, including high cost, limited materials, insufficient precision, etc. How to consider and solve these problems in this article?

Response:

We fully agree to your comment. Our publication is intended as a guideline to test printers, technology and materials to simplify the application for non-expert users. However, as every set-up and every printer is different, everyone will have to adjust the process for the own needs and purposes to overcome these limitations (116-130, 546-556).

We can give assistance to choose the best printing method or material (Fig 5), but we can not solve these fundamental problems in this paper.

We hope that we could respond to the comment sufficiently.

4) All figures need to be modified for higher quality.

Response:

Thank you for highlighting this problem for us. We have checked our figures using the Preflight Analysis and Conversion Engine (PACE) und uploaded the respective figure files with higher quality.

5) The quality of English needs improving. The manuscript should be concise and emphasize the most important points of your work.

Response:

Thank you for indicating problems in terms of the language. We had an additional check for the quality of English by a professional English-speaking person, who we also added to the acknowledgements section. All changes made are highlighted in the revised manuscript.

6) Most of the references are relatively old, I recommend the authors to cite new references in recent years.

Response:

Thank you for raising this issue. We carefully revised our literature references and added more relevant and recent ones.

For most of the older references, we couldn’t think of an adequate replacement. In the introduction, we explain the term Tissue Engineering. As this term was introduced more than 30 years ago, there are many older publications explaining and defining this field. Publications that are more recent do not explain this issue in detail and refer to these older publications. Furthermore, we give examples for tissue engineered substitutes and applications of 3D printing in medicine, which have already been achieved. Therefore, we cite the original publications, which is the common and good practice.

All in all, more than 50 % of our references were published between 2016 and 2020.

We hope that we were able to address the reviewer’s comment adequately.

We thank the reviewers for the thorough evaluation of the manuscript and the constructive comments.
